# The Progress of Emergency Medicine in Taiwan, China, and Hong Kong: Perspective from Publications in Emergency Medicine Journals, 1992–2011

**DOI:** 10.1155/2014/682375

**Published:** 2014-02-23

**Authors:** Ching-Hsing Lee, Chung-Hsien Chaou, Chih-Chuan Lin

**Affiliations:** ^1^Department of Emergency Medicine, Chang Gung Memorial Hospital, Keelung and Chang Gung University College of Medicine, Keelung 204, Taiwan; ^2^Department of Emergency Medicine, Chang Gung Memorial Hospital, Linkou and Chang Gung University College of Medicine, Taoyuan 333, Taiwan

## Abstract

*Study Objective*. The progress of emergency medicine (EM) in Taiwan, China, and Hong Kong was evaluated from the perspective of publications in EM journals. *Methods*. This was a retrospective study. All articles published from 1992 to 2011 in all journals in the EM category in the 2010 Journal Citation Reports (JCR) were included. A computerized literature search was conducted using the SciVerse Scopus database. The slope (**β**) of the linear regression was used to evaluate the trends in the numbers of articles as well as the ratios to the total number of EM journal articles. *Results*. The trends in the numbers of articles from Taiwan, China, and Hong Kong were 6.170, 1.908, and 2.835 and the trends in the ratios of their publication numbers to the total number of EM journal articles were 15.0 × 10^−4^, 4.60 × 10^−4^, and 6.80 × 10^−4^, respectively. All *P*-values were <0.01. The mean, median, and 75th percentiles of the number of citations in all EM journals were greater than those of these three areas. *Conclusions*. The publications from Taiwan, China, and Hong Kong have increased at a higher rate than those of the overall EM field in the past 20 years and indicated the rapid progress in these three areas.

## 1. Introduction

Emergency medicine (EM) is a rapidly developing medical specialty that originated in the 1970s [1, 2]. The development of EM has been reported worldwide and has been evaluated from many perspectives [3–5]. Most studies adopted the founding year, such as the founding of national organizations, nationally recognized medical specialty, board certification, standardized residency training, and specialty journals, as a milestone to evaluate the progress of EM [6–8]. These indicators are acceptable and are key components of the EM system but do not provide a quantitative, qualitative, and temporal trend evaluation of the progress of EM. During development of a medical specialty, academic research, article number, and specialty journals mature as the scope and size of the medical field grow [9]. Thus, the trend in scientific articles may provide a quantitative and dynamic assessment of the development of a medical specialty.

Taiwan, China, and Hong Kong are close in terms of geographic location, culture, and language. The milestones of EM development in these areas occurred at similar times. The first national organizations in these areas were all founded in the 1980s. The Society of Emergency & Critical Care Medicine (SECCM) and the Taiwan Society of Emergency Medicine (TSEM) were founded in 1982 and 1994, respectively [10, 11]. In China, the Chinese Society of Emergency Medicine (CSEM) and the Chinese College of Emergency Physicians were founded in 1987 and 2009, respectively [12–14]. In Hong Kong, the Hong Kong Society for Emergency Medicine & Surgery and the Hong Kong College of Emergency Medicine (HKCEM) were founded in 1985 and 1996, respectively [15, 16]. EM became a nationally recognized medical specialty and a certification board was formed in Taiwan and Hong Kong in 1997 [11, 16]. These milestones provide a general understanding of EM development in these areas in the 1990s but do not portray its more recent and dynamic evolution. Various landmarks in recent years indicate the rapid progress of EM in these areas. The official journal of the HKCEM, the *Hong Kong Journal of Emergency Medicine* (HKJEM), was enrolled in the 2010 Journal Citation Reports (JCR) Science Edition [17]. Authors from Taiwan and China were the first and sixth contributors outside the United States to submit manuscripts to *Annals of Emergency Medicine*, one of the leading EM journals in JCR category of EM [18]. Evaluation of EM progress and related studies in these areas has to date been limited. The goal of this investigation was to provide a quantitative and dynamic evaluation of the multidimensional evolution of EM and EM studies in Taiwan, China, and Hong Kong by analyzing the trends and characteristics of scientific articles published in EM journals.

## 2. Methods

### 2.1. Study Design

This study was retrospective and qualified for a waiver from our institutional review board because no human subjects were involved.

### 2.2. Study Setting and Population

The study period was 1992–2011. A list of EM journals was collected from the EM category in the 2010 JCR Science Edition. The details of published articles were collected from the SciVerse Scopus database. The websites of the EM journals were accessed if the SciVerse Scopus database did not include articles published within the inclusion years investigated. All journals included in the EM category in the 2010 JCR were included as source journals. The article inclusion year of each source journal was determined by its year of inclusion in the JCR. For journals included in the JCR before 1992, all articles between 1992 and 2011 were enrolled. For journals included in the JCR after 1992, the article inclusion year was 2 years before the year of inclusion of the journal in the JCR, to 2011. This adjustment was performed for reasons of consistency with the definition and calculation of impact factor in the JCR [19]. All articles published within the inclusion years of each journal were included. Articles originating from Taiwan, China, and Hong Kong were classified according to the affiliations of the first and corresponding authors. If both the first and corresponding authors were not from these areas, the article was classified as not having originated from Taiwan, China, or Hong Kong. In-press publications were excluded.

### 2.3. Study Protocol

#### 2.3.1. Articles in EM Journals

The list of source journals and their International Standard Serial Numbers (ISSN) were retrieved from the EM category in 2010 JCR. A computerized literature search was conducted using the SciVerse Scopus database on March 17, 2012. The search terms used were “ISSN (xxxx-xxxx) AND PUBYEAR AFT yyyy AND PUBYEAR BEF 2012” (The “xxxx-xxxx” refers to the ISSN of the journal and “yyyy” refers to the year immediately preceding the article inclusion year). The list of source journals, year of journal inclusion in the JCR, and the article inclusion year are shown in Table 1. The search terms for articles in each journal are shown in The Appendix. *Emergencias* and *Signa Vitae* were enrolled in the JCR in 2010 and 2008. The SciVerse Scopus database included articles from these journals in the same years. Articles in *Emergencias* in 2008 and 2009 and those in *Signa Vitae* in 2006 and 2007 were retrieved from the websites of the journals [20, 21].

#### 2.3.2. Articles Originating from Taiwan, China, and Hong Kong

Articles originating from Taiwan, China, and Hong Kong in each EM journal were retrieved by adding the search terms “AND AFFILCOUNTRY (Taiwan),” “AND AFFILCOUNTRY (China),” and “AND AFFILCOUNTRY (Hong Kong)” to the search term of each EM journal. The articles retrieved were reviewed by one of the authors to confirm that they were classified according to the affiliations of the first and corresponding authors. Articles were excluded if both the first and corresponding authors were not from these areas.

We collected data on the journal, year, publication type, and the number of times cited for all articles included in the analysis. Data were analyzed using the SAS statistical software, version 9.2 (SAS Institute, Cary, NC, USA).

### 2.4. Measurements

The numbers of articles published in the EM journals were adopted as the quantitative evaluation of academic performance in EM. The primary outcome measures were the trends in article numbers in EM journals originating from Taiwan, China, and Hong Kong and in the ratio to the total number of articles in all 1992–2011 EM journals. The trends between 2002 and 2011 were also calculated to evaluate evolution over the past 10 years.

Secondary outcome measures were the article distributions in EM journals, the changes in the numbers of articles published in each EM journal, the distribution of the number of times an article was cited, and the proportion of each article type.

### 2.5. Data Analysis

Linear regression was used to evaluate the trends in the numbers of articles from Taiwan, China, and Hong Kong and in the ratio to the total number of articles in all EM journals. The slope (*β*) of the linear regression was adopted as a representative of the trend. The 95% confidence intervals of *β* were calculated. The distribution of articles in EM journals, number of citations, and publication types were analyzed using descriptive statistics. A value of *P* < 0.05 was considered to indicate statistical significance.

## 3. Results

**Figure 1:**
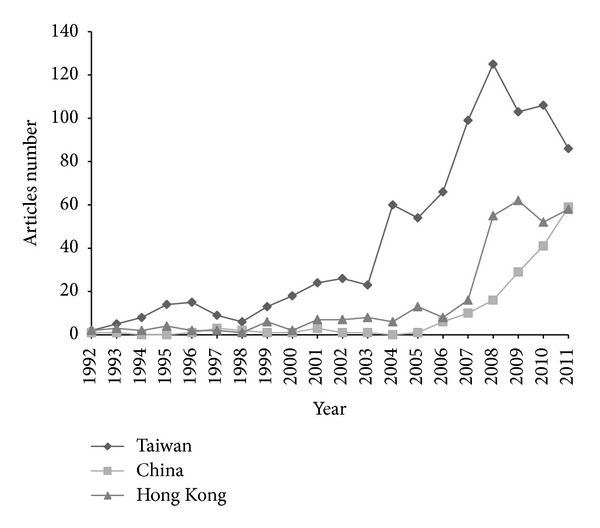
Numbers of articles from Taiwan, China, and Hong Kong published in emergency medicine journals from 1992 to 2011.

**Figure 2:**
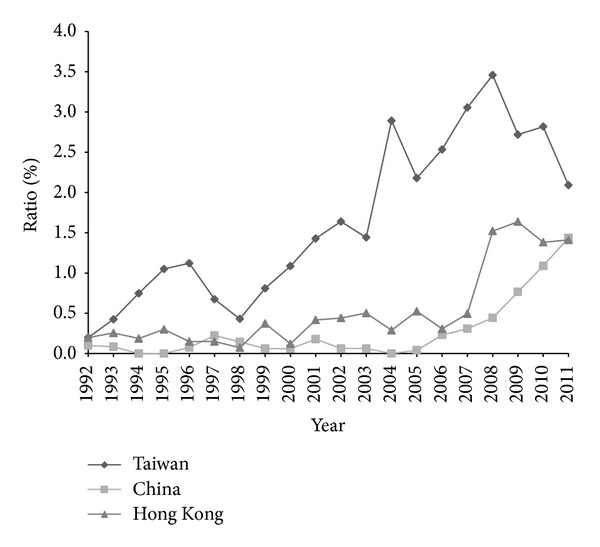
Ratios of the numbers of articles from Taiwan, China, and Hong Kong to the total number of emergency medicine journal articles published from 1992 to 2011.

Twenty-three journals were classified in the EM category in the 2010 JCR. The numbers of articles from Taiwan, China, and Hong Kong published in each EM journal during the study period are shown in Table 1 and Figure 1. Several turning points after 2008 were noted. The number of articles from Hong Kong increased rapidly in 2008, whereas the number from Taiwan decreased, and the gaps between Taiwan and China and Taiwan and Hong Kong diminished. The number of articles from China surpassed that from Hong Kong in 2011. The number of articles from Taiwan and Hong Kong grew gradually during the study period, but China showed a rapid rising trend since 2006. The ratios of article numbers to the total number of articles in all EM journals are shown in Figure 2. The trends (*β*) in the numbers of articles from Taiwan, China, and Hong Kong and in the ratios to the total published number of all articles in all EM journals from 1992 to 2011 and from 2002 to 2011 are shown in Table 2. The number of articles in EM journals from Taiwan, China, and Hong Kong increased at a faster rate during these two periods than did the total number of EM journal articles. These trends were all statistically significant, with the exception of that in the ratio of the number of articles from Taiwan to the total in all EM journals between 2002 and 2011. The rate of increase in the number of articles from Taiwan was greater than that of those from China and Hong Kong between 1992 and 2011; however, the difference was not significant between 2002 and 2011.

**Figure 3:**
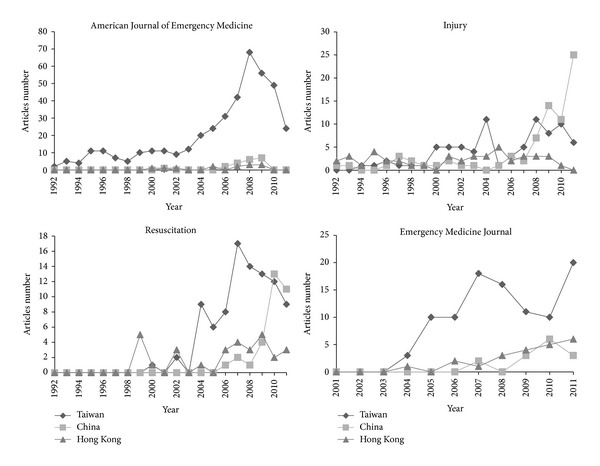
Numbers of articles from Taiwan, China, and Hong Kong in the four emergency medicine journals in which they were published most commonly.

The numbers of articles in EM journals from Taiwan, China, and Hong Kong were not distributed evenly among the EM journals (Table 3). Two (8.7%) of the 23 EM journals published nearly half (647/1355, 47.7%) of all articles from these three areas. Seven EM journals (7/23, 30.4%) published 91.3% (1237/1355) of all articles. These seven journals were the *American Journal of Emergency Medicine* (445, 32.8%), *Injury* (202, 14.9%), *HKJEM* (160, 11.8%), *Resuscitation* (153, 11.3%), *Emergency Medicine Journal* (134, 9.9%), *Journal of Emergency Medicine* (76, 5.6%), and *Annals of Emergency Medicine* (67, 4.9%). The numbers of articles from Taiwan, China, and Hong Kong in the four EM journals in which they were published most commonly are shown in Figure 3. The numbers of articles in *HKJEM* were not plotted because only four publication years were included.

The mean, standard deviation, 25th percentile, median, 75th percentile, mode, maximum, skewness, and kurtosis of the number of citations from Taiwan, China, and Hong Kong and all EM journal articles are shown in Table 4. The publication types in the SciVerse Scopus database were classified as Article, Review, Letter, Editorial, Short Survey, Note, Erratum, and Conference papers. The proportions of each publication type from Taiwan, China, and Hong Kong and in all EM journals are shown in Table 5.

## 4. Discussion

This study focused mainly on articles in EM journals from the JCR. In addition to the JCR journals, EM-related studies are also published in national EM specialty journals not included in JCR. A brief review of the official journals of the national EM specialty organizations of Taiwan, China, and Hong Kong provided another perspective of the progress of EM and a comparison with our results. The *Journal of Emergency and Critical Care Medicine* (*JECCM*), the official journal of SECCM, was first published in 1990 [22]. The *Journal of Taiwan Emergency Medicine* (*JTEM*), the official journal of TSEM, was first published in 1999 [23]. The *JECCM* and *JTEM* are published in English. Thirteen EM-related journals are available in China [24]. The *Chinese Journal of Emergency Medicine*, the official journal of CSEM, was first published in 1990 [25]. The *World Journal of Emergency Medicine*, the only English EM specialty journal in China, was first published in 2010 [26]. The *HKJEM*, the official journal of HKCEM, was first published in 1994 in English and is the only EM specialty journal included in JCR in these three areas [17]. The publication of a national EM specialty journal serves as a milestone of academic research at the national level [27, 28]. The first official journals of national EM specialty organizations in these areas were all first published in the 1990s, regardless of the publication language. The journal publication language is one of the factors that can influence journal internationalization [29]. English has become the international language for scientific studies and allows research results to be seen by most international researchers [29, 30]. The first English EM-specialty journals in Taiwan and Hong Kong were first published 20 and 16 years, respectively, earlier than those in China. This timing is compatible with our finding that the increase in the number of scientific articles published in JCR EM journals from Taiwan and Hong Kong was both earlier and faster than that of articles from China. The first English EM-specialty journal in China was first published in 2010. This timing is compatible with the recent rapid increase in scientific articles from China published in JCR EM journals. The results indicate that EM research in China has been progressing rapidly in recent years. Authors from Taiwan and Hong Kong have contributed many articles to JCR EM journals in the past few decades. The *HKJEM* was enrolled in the JCR in 2010; however, no EM-specialty journal originating from Taiwan is included in the JCR. In addition, the number of articles from Taiwan published in EM journals has decreased since 2008. These results should be considered by both the national organizations of, and emergency physicians in, Taiwan.

Articles from Taiwan, China, and Hong Kong are concentrated in a limited number of journals, and some journals publish studies from these three areas only rarely. About 80% of the articles from these three areas are concentrated in five journals. These journals were in English, originated from the United States and Europe, had a journal impact factor rank in the top half of the EM category, and were included in the JCR before 1992. Thirteen journals published fewer than 10 articles from these three areas and were not published in English (German, Turkish, or Spanish), originated outside the United States, and were enrolled in the JCR after 2005 or were invitation-only journals. This phenomenon may have been due to three reasons. First, the longer the journal had been included in the JCR, the more publications were included. Second, the number of EM journals in the JCR expanded rapidly, such that the distribution of articles inevitably became uneven. The EM category in the JCR was independent in 2000 and included only 12 journals. Eight of the 12 journals remained in the category, and 23 were included in the EM category of the 2010 JCR [31]. Third, the most common foreign language in these three areas is English, so English journals are the first to which articles are submitted. This indicates that EM is a rapidly growing specialty and informs emergency physicians of the progress of EM research in non-English speaking countries, newly published EM journals, and outside the United States.

The trend in the numbers of articles from these three areas published in each EM journal revealed the major contributors to the turning points in 2008–2011. The number of articles from Hong Kong increased rapidly in 2008, which was attributable mainly to inclusion of articles in *HKJEM* since 2008, and this number plateaued. The decrease in the number of articles from Taiwan since 2008 resulted mainly from the decrease in the number of articles published in the *American Journal of Emergency Medicine* and *Resuscitation*. The increase in the number of articles from China was contributed to primarily by *Resuscitation* and *Injury*, which are leading journals in the EM category of the JCR. Thus the progress of EM and related research in China is undergoing not only a quantitative but also a qualitative change. The reasons for the decrease in the number of articles from Taiwan and the plateau in the number from Hong Kong since 2008 require further study.

The number of times an article is cited represents the degree of its influence on other publications and was adopted in this study as an indicator of the impact and quality of articles [32]. The distributions of cited times of an article from these three areas and all EM journals were skewed but exhibited a pattern identical to those of scientific publications in other fields [19]. The maximum, 75th percentile, median, and mean of the number of citations in all EM journals were higher than those of these three areas individually. The 75th percentile, median, and mean number of citations for articles from Taiwan were higher than those of articles from China and Hong Kong. This distribution indicates that the average influence of all EM journal publications was greater than those of these three areas individually and that the mean influence of Taiwanese articles was greater than that of articles from China and Hong Kong. An evaluation of the number of times an article was cited may not immediately reflect the influence of recent publications due to the time lag between the cited publication and the citing article. The proportions of recent publications from these three areas were higher than those of all EM journals. In addition to quantitative change in recent years, whether there is also qualitative change in these areas from the perspective of the numbers of times cited requires further study.

The majority of publications were “Article,” the proportions of which from these three areas were higher than those of all EM journals. The proportion of “Editorials” was lower in Taiwan and China than in Hong Kong. This may be explained by the fact that no JCR journal originates from Taiwan or China, and the origin of the Editorial tends to be identical to that of the journal.

## 5. Limitations

There are limitations of this study that should be mentioned. First, the development of EM is a multidimensional process. The trend in scientific publications in EM journals provides only one of the multiple aspects of the whole picture, in addition to other milestones. No single method can evaluate overall progress comprehensively. Second, EM is closely related to many other medical fields, and the impact of EM-related studies published in non-EM JCR journals was not evaluated. Third, the trends mentioned in this paper may not be purely linear. However, Quadratic forms were not used for its difficulty in interpretation. Fourth, the evolution of a medical specialty is a complex process and is influenced by many factors [27]. The forces driving this evolution were not investigated here.

## 6. Conclusions

In the past 20 years, the numbers of articles from Taiwan, China, and Hong Kong published in EM journals increased substantially and exceeded the overall rate of increase in the total number of EM journal articles. This result confirms the rapid progress of EM in these three areas in addition to milestones of EM evolution. The quantity and quality of articles that originated from Taiwan were greater than those from China and Hong Kong. However, the gaps between Taiwan and China and Hong Kong have diminished since 2008.

## Figures and Tables

**Table 1:** Numbers of articles from Taiwan, China, and Hong Kong published in each of the 23 emergency medicine journals from 1992 to 2011 in the 2010 Journal Citation Reports.

Title of Journal*	ISSN	Language	Journal inclusion year in JCR	Articles inclusion year	Articles number from Taiwan	Articles number from China	Articles number from Hong Kong	Total article number
Resuscitation	0300-9572	English	before 1992	1992	91	32	30	3969
Annals of Emergency Medicine	0196-0644	English	before 1992	1992	53	3	11	6606
Emergencias	1137-6821	Spasnish and English	2010	2008	0	1	0	455
Injury	0020-1383	English	before 1992	1992	81	77	44	5074
Academic Emergency Medicine	1069-6563	English	1997	1995	23	1	2	3973
Scandinavian Journal of Trauma, Resuscitation and Emergency Medicine	1757-7241	English	2010	2008	0	0	0	209
American Journal of Emergency Medicine	0735-6757	English	before 1992	1992	412	20	13	4629
Prehospital Emergency Care	1090-3127	English	2008	2006	0	0	0	466
Journal of Emergency Medicine	0736-4679	English	2001	1999	61	6	9	2710
Canadian Journal of Emergency Medicine	1481-8035	English	2010	2008	0	0	1	327
Emergency Medicine Clinics of North America	0733-8627	English	2000	1998	0	0	0	826
Emergency Medicine Journal	1472-0205	English	2002	2000	98	14	22	3046
Emergency Medicine Australasia	1742-6731	English	2009	2007	3	0	6	529
European Journal of Emergency Medicine	0969-9546	English	2009	2007	4	0	19	422
Pediatric Emergency Care	0749-5161	English	1994	1992	23	6	6	3082
Unfallchirurg	0177-5537	German	before 1992	1992	0	0	0	2980
Journal of Emergency Nursing	0099-1767	English	2007	2005	2	1	0	1116
Notfall und Rettungsmedizin	1434-6222	German	2009	2007	0	0	0	500
European Journal of Trauma and Emergency Surgery	1863-9933	English	2009	2007	2	5	0	471
Ulusal Travma ve Acil Cerrahi Dergisi	1306-696X	Turkish	2009	2007	1	3	0	479
Signa Vitae	1334-5605	English	2008	2006	7	2	0	97
Hong Kong Journal of Emergency Medicine	1024-9079	English	2010	2008	1	6	153	284
Notarzt	0177-2309	German	2009	2007	0	0	0	207
Total					**862**	**177**	**316**	**42457**

*Titles are sorted according to impact factor of 2010 JCR.

**Table 2:** Trends (*β*) in the numbers of publications from Taiwan, China, and Hong Kong and in the ratios to the total number of emergency medicine publications from 1992 to 2011 and from 2002 to 2011.

	Trend (*β*)	95% CI*	*P* value
Numbers of publications (1992 to 2011)			
Taiwan	6.170	4.685 to 7.654	<0.001
China	1.908	0.959 to 2.856	0.001
Hong Kong	2.835	1.720 to 3.949	<0.001
The ratios to the total number of EM publications (1992 to 2011)			
Taiwan	15.0 × 10^−4^	11.1 × 10^−4^ to 18.9 × 10^−4^	<0.001
China	4.60 × 10^−4^	2.27 × 10^−4^ to 6.93 × 10^−4^	0.001
Hong Kong	6.80 × 10^−4^	4.06 × 10^−4^ to 9.54 × 10^−4^	<0.001
Numbers of publications (2002 to 2011)			
Taiwan	9.588	4.503 to 14.673	0.02
China	6.036	3.658 to 8.415	<0.001
Hong Kong	7.158	4.015 to 10.300	0.001
The ratios to the total number of EM publications (2002 to 2011)			
Taiwan	1.04 × 10^−3^	−0.45 × 10^−3^ to 2.53 × 10^−3^	0.146
China	1.49 × 10^−3^	0.94 × 10^−3^ to 2.04 × 10^−3^	<0.001
Hong Kong	1.53 × 10^−3^	0.621 × 10^−3^ to 2.44 × 10^−3^	0.005

*Confidence interval.

**Table 3:** The five emergency medicine journals in which articles from Taiwan, China, and Hong Kong were published most commonly.

Rank	Taiwan (N*, %)	China (*N*, %)	Hong Kong (*N*, %)
1	AJEM (412, 47.8%)	Injury (77, 43.5%)	HKJEM (153, 48.4%)
2	EMJ (98, 11.4%)	Resuscitation (32, 18.1%)	Injury (44, 13.9%)
3	Resuscitation (91, 10.6%)	AJEM (20, 11.3%)	Resuscitation (30, 9.5%)
4	Injury (81, 9.4%)	EMJ (14, 7.9%)	EMJ (22, 7.0%)
5	JEM (61, 7.1%)	JEM (6, 3.4%)	EJEM (19, 6.0%)

*Publication number.

AJEM: American Journal of Emergency Medicine; EMJ: Emergency Medicine Journal; JEM: Journal of Emergency Medicine; HKJEM: Hong Kong Journal of Emergency Medicine; EJEM: European Journal of Emergency Medicine.

**Table 4:** Distribution of cited times of articles from Taiwan, China, Hong Kong and all emergency medicine journals.

Cited times	Taiwan	China	Hong Kong	All EM journals
Mean	4.561	2.633	3.775	8.045
Standard deviation	7.687	5.259	9.395	17.094
25 percentile	0	0	0	0
Median	2	1	0	2
75 percentile	5	3	2.25	9
Mode	0	0	0	0
Maximun	50	44	80	940
Skewness	2.96	4.35	4.49	10.11
Kurtosis	10.23	25.73	24.81	288.86

**Table 5:** Proportions of types of publication from Taiwan, China, and Hong Kong and those in all emergency medicine journals.

Publication type	Taiwan	China	Hong Kong	All EM journals
Article	78.5%	84.2%	72.5%	66.1%
Review	1.7%	3.4%	5.4%	9.3%
Letter	17.2%	11.9%	11.1%	11.2%
Editorial	0.2%	0.6%	7.9%	4.8%
Short Survey	1.4%	0.0%	0.9%	2.2%
Note	0.8%	0.0%	2.2%	3.5%
Erratum	0.1%	0.0%	0.0%	1.1%
Conference papers	0.0%	0.0%	0.0%	1.7%

**Table 6:** 

Title of Journal*	ISSN	Search term
Resuscitation	0300-9572	ISSN (0300-9572) AND PUBYEAR AFT 1991 AND PUBYEAR BEF 2012
Annals of Emergency Medicine	0196-0644	ISSN (0196-0644) AND PUBYEAR AFT 1991 AND PUBYEAR BEF 2012
Emergencias	1137-6821	ISSN (1137-6821) AND PUBYEAR AFT 2007 AND PUBYEAR BEF 2012
Injury	0020-1383	ISSN (0020-1383) AND PUBYEAR AFT 1991 AND PUBYEAR BEF 2012
Academic Emergency Medicine	1069-6563	ISSN (1069-6563) AND PUBYEAR AFT 1994 AND PUBYEAR BEF 2012
Scandinavian Journal of Trauma, Resuscitation and Emergency Medicine	1757-7241	ISSN (1757-7241) AND PUBYEAR AFT 2007 AND PUBYEAR BEF 2012
American Journal of Emergency Medicine	0735-6757	ISSN (0735-6757) AND PUBYEAR AFT 1991 AND PUBYEAR BEF 2012
Prehospital Emergency Care	1090-3127	ISSN (1090-3127) AND PUBYEAR AFT 2005 AND PUBYEAR BEF 2012
Journal of Emergency Medicine	0736-4679	ISSN (0736-4679) AND PUBYEAR AFT 1998 AND PUBYEAR BEF 2012
Canadian Journal of Emergency Medicine	1481-8035	ISSN (1481-8035) AND PUBYEAR AFT 2007 AND PUBYEAR BEF 2012
Emergency Medicine Clinics of North America	0733-8627	ISSN (0733-8627) AND PUBYEAR AFT 1997 AND PUBYEAR BEF 2012
Emergency Medicine Journal	1472-0205	ISSN (1472-0205) AND PUBYEAR AFT 1999 AND PUBYEAR BEF 2012
Emergency Medicine Australasia	1742-6731	ISSN (1742-6731) AND PUBYEAR AFT 2006 AND PUBYEAR BEF 2012
European Journal of Emergency Medicine	0969-9546	ISSN (0969-9546) AND PUBYEAR AFT 2006 AND PUBYEAR BEF 2012
Pediatric Emergency Care	0749-5161	ISSN (0749-5161) AND PUBYEAR AFT 1991 AND PUBYEAR BEF 2012
Unfallchirurg	0177-5537	ISSN (0177-5537) AND PUBYEAR AFT 1991 AND PUBYEAR BEF 2012
Journal of Emergency Nursing	0099-1767	ISSN (0099-1767) AND PUBYEAR AFT 2004 AND PUBYEAR BEF 2012
Notfall und Rettungsmedizin	1434-6222	ISSN (1434-6222) AND PUBYEAR AFT 2006 AND PUBYEAR BEF 2012
European Journal of Trauma and Emergency Surgery	1863-9933	ISSN (1863-9933) AND PUBYEAR AFT 2006 AND PUBYEAR BEF 2012
Ulusal Travma ve Acil Cerrahi Dergisi	1306-696X	ISSN (1306-696X) AND PUBYEAR AFT 2006 AND PUBYEAR BEF 2012
Signa Vitae	1334-5605	ISSN (1334-5605) AND PUBYEAR AFT 2005 AND PUBYEAR BEF 2012
Hong Kong Journal of Emergency Medicine	1024-9079	ISSN (1024-9079) AND PUBYEAR AFT 2007 AND PUBYEAR BEF 2012
Notarzt	0177-2309	ISSN (0177-2309) AND PUBYEAR AFT 2006 AND PUBYEAR BEF 2012

*Titles are sorted according to impact factor of 2010 JCR.

## Appendix

For more details see Table 6.
